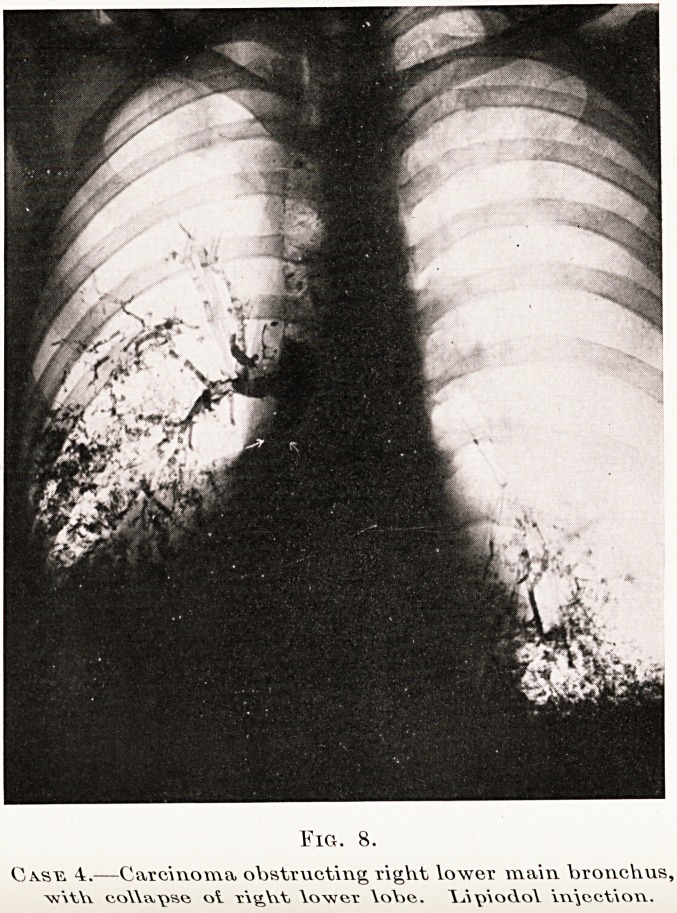# Primary Carcinoma of the Lung Radiologically Considered

**Published:** 1936

**Authors:** Gilbert B. Bush

**Affiliations:** Hon. Assistant Radiologist to the Bristol General Hospital; Hon. Radiologist to the General Hospital, Weston-super-Mare


### CARCINOMA OF THE BRONCHUS.

### Primary Carcinoma of the Lung Radiologically Considered.

By GILBERT B. BUSH, M.B., Ch.B., D.M.R.E.,

*Hon. Assistant Radiologist to the Bristol General Hospital; Hon. Radiologist to the General Hospital, Weston-super-Mare.*

It is generally admitted that the routine X-ray examination of the chest in all cases presenting doubtful signs and symptoms has during the past ten years or so contributed materially to the earlier recognition of this tragic disease of the lungs, but it it still unfortunately true that in the majority of cases which come for radiological investigation the disease is generally too far advanced for other than palliative treatment.

The radiographic features in this disease closely follow and are dependent upon the pathological process taking place, and the stage of development and its secondary results will determine the X-ray findings in a particular case.

I propose, therefore, briefly to follow the stages of the disease from its earliest beginnings, and correlate the morbid anatomy and physiological functional changes with the abnormal radiological appearances as observed on fluoroscopic examination and radiographs. If the pathological processes are fully appreciated, it is thus possible to argue back from the X-ray findings in the majority of cases, and glean a considerable amount of useful information as to the nature, extent and stage of the disease when one meets it. The X-ray examination will also, in many cases, indicate what lines of treatment are possible, and assist in the prognosis.

### Section I.

*When the disease is confined to the Lung.*

1. *Pathological changes.*—The growth is in the endobronchial stage, with a small tumour and incomplete obstruction. The obstruction is often more marked during expiration than during inspiration.

*X-ray findings.*—There may be none. But one may find delayed or incomplete deflation in the affected lobe or lobule if films are taken on inspiration and expiration. Lipiodol injection may show a filling defect, particularly if serial films are made. Lenk has also described a peculiar "mediastinal jerking," observed on screen examination.

2 (*a*). *Pathological changes.*—The bronchus becomes occluded, producing collapse or deflation of a lobe; if in a main bronchus, then of one lung. At this stage the growth has generally eroded through the bronchus, and the neighbouring tissue and lymph-nodes are affected.

*X-ray findings.*—(Lobar manifestations.)

(*a*) There is opacity in the area supplied by the bronchus, generally a lobe. (Fig. 1, Case 2.) The whole of the lung may be opaque if the main bronchus is obstructed. (Fig. 2.)

If films are made with a penetrating ray and the Bucky diaphragm, the shadow is seen to have two components, a distal cloudy zone, showing no striations, like ground-glass, which is deflated lung, and a denser central zone merging into the mediastinal shadow and showing vascular striations. This denser shadow

### PLATE IX

Diagram A.

Diagram B.

Diagram C.

Diagram D.

PLATE X

Fig. 1.

Case 2.—Carcinoma of right upper lobe bronchus, with deflation of upper lobe, and phrenic paralysis.

Fig. 2.

Case 3.—Carcinoma of right main bronchus. Deflation of whole right lung. Tracheal "swing" to the right. Secondary infection in left upper lobe.

Fig. 3.

To illustrate the type of tracheal displacement in lung fibrosis (chronic fibroid phthisis).

Fig. 4.

Case 1.—Carcinoma of first axillary branch of left upper lobe bronchus, with involvement of paraortic glands and recurrent laryngeal nerve. (July, 1935.)

Fig. 5.

Child, aged 6, with lymphadenitis of the tracheo-bronchial glands, with surrounding lung-hyperæmia.

(Figure is reversed right to left).

Fig. 6.

Case 1.—September, 1935. Growth extending. Glands larger.

is growth, but the large blood-vessels near the hilum are not obliterated. (Diagram A.)

(*b*) The *interlobar fissures* are altered in position. (Fig. 1 and Diagram A.) Antero-posterior or lateral films will usually show this.

(*c*) If the stenosis is in a bronchus of the first degree the *mediastinum and trachea are displaced* to the side of the lesion, and this displacement is seen on screen examination to be increased during inspiration. Kerley points out that the type of tracheal displacement can give a valuable clue to the nature of the pathological process underlying a solid-looking lung, and will often differentiate a displacement due to collapse from that due to fibrosis. In the former (Fig. 2) the trachea tends to swing as a whole towards the side of the lesion, whereas in fibrosis (Fig. 3) it bends towards that side at about the level of the sternal notch.

*Differential diagnosis.*—A similar general appearance may be produced by (1) a foreign body lodged in a bronchus, (2) post-operative collapse, (3) pneumonic infection with collapse, (4) silicosis producing stenosis of a bronchus. A Potter-Bucky picture will help to exclude these conditions, while the mode of onset of symptoms, history and clinical findings should even more definitely assist one in making a diagnosis.

2 (*b*). *Pathological changes.*—No bronchial stenosis, but the growth may rapidly erode through the bronchial wall and infiltrate the lung.

*X-ray findings.*—This produces the "nodular" type of shadow in the lung-field, most commonly in the hilar zone. (Fig. 4, Case 1, and Diagram B.) Being placed deep in the lung, it may produce no physical signs, although symptoms may be suggestive. One sees a fairly dense opacity around the root of the lung with no collapse or consolidation in the more distal parts of the lobe or lung as a whole. The shadow has a poorly-defined outer border, and generally shows linear shadows radiating out into the lung. Also lung-markings are visible in the opacity. Enlargement of the tracheo-bronchial glands can sometimes be demonstrated. At any time this may pass into the lobar type already described.

*Differential diagnosis.*—From (1) lymphadenoma infiltrating the lung. Here other glands may be involved in the neck, axilla, etc., and paratracheal glands are often enlarged ; also the lesions are generally bilateral in the chest. From (2) lymphadenitis of the tracheo-bronchial glands, with surrounding lung-hyperæmia (Fig. 5). Here the age of the patient is important, as this condition is generally found in children (so-called "hilum tuberculosis"). (3) Lastly, some rare forms of pulmonary syphilis (Kerley).

3. *Pathological changes.* — Infection supervenes distal to the bronchial obstruction with bronchiectasis, necrosis, abscess-formation and possibly gangrene.

*X-ray findings.*—Clearer areas with fluid levels may be seen within the tumour shadow. (Diagram D.) If the patient is first seen at this stage we have to differentiate from a non-malignant lung abscess. (Diagram C.) In the malignant type the denser part of the shadow is nearly always continuous with that of the hilum, and the breaking-down area is eccentrically placed in the shadow.

4. *Pathological changes.*—Mucoid degeneration may occur in the tumour.

*X-ray findings.*—Kerley has reported two cases in which this was detected and demonstrated radioscopically, the tumour appearing to "shake like a jelly."

PLATE XIII

Fig. 7.

Case 1.—March, 1936. Growth and glands further enlarged. Early deflation of part of upper lobe. Left phrenic paralysis.

Fig. 8.

Case 4.—Carcinoma obstructing right lower main bronchus, with collapse of right lower lobe. Lipiodol injection.

### SECTION 2.

### *Secondary Changes outside the Lung.*

If any definite signs of these (see below) are found radical removal of the growth by lobectomy is contra-indicated.

1. *Pathological changes.*—Lymph-glands at the root of the lung are infected by growth.

*X-ray findings.*—Small bronchial glands are generally obscured by overlying growth, as are also the tracheo-bronchial glands, though the latter can sometimes be seen. (Figs. 4, 6 and 7, Case 1.)

2. *Pathological changes.*—Compression or infiltration of the phrenic nerve, producing paralysis of one half of the diaphragm.

*X-ray findings.*—The diaphragm on the affected side is raised, and shows "paradoxical movement" on inspiration. (Figs. 1 and 7.) The commonest cause of this finding is neoplasm, but an aortic aneurysm of long standing can sometimes produce it.

3. *Pathological changes.*—Involvement of the vagus, or secondary deposits in the abdomen, producing gastro-intestinal symptoms.

*X-ray findings.*—Cases referred for gastric ulcer or carcinoma of the digestive tract sometimes turn out to be primary carcinoma of the lung. The chest should always be screened as a routine in abdominal cases.

4. *Pathological changes.*—Pleural involvement, with effusion.

*X-ray findings.*—Here the dense shadow of the effusion obscures everything in the lung. Paracentesis and further examination sometimes helps, but indirect X-ray signs may be obtained. For instance, if there are signs of a large effusion, and yet the heart and mediastinum are not displaced, something else is underlying the effusion, either collapse or fibrosis. The type of displacement of the trachea will help to decide which.

5. *Pathological changes.*—There may be a very small primary growth in a bronchus which rapidly invades a blood-vessel, producing one or more secondary nodules or masses in the lung.

*X-ray findings.*—There is no hilar shadow, but single or multiple isolated shadows are seen in the lung. In this case one cannot be sure where the primary is. Some think that this is a form of primary alveolar carcinoma, but the question is debatable, and one for pathologists to settle.

### SECTION 3.

### *Accessory Methods of Examination.*

1. *Lipiodol injection.* (Fig. 8.)—I have only time merely to mention this. In selected cases it is a most valuable accessory method to demonstrate obstruction or filling defects in the bronchi, particularly if serial studies are made. If expert bronchoscopy is available this may be preferable.

2. *Artificial pneumothorax.*—This is sometimes useful in order to differentiate an intrapulmonary from an extrapulmonary tumour.

In conclusion, I would like to thank various colleagues for referring patients for examination, and particularly to Dr. Peter Kerley, of London, for the loan of some of the slides and for much valuable help and advice in preparing these notes.

## Figures and Tables

**Diagram A. f1:**
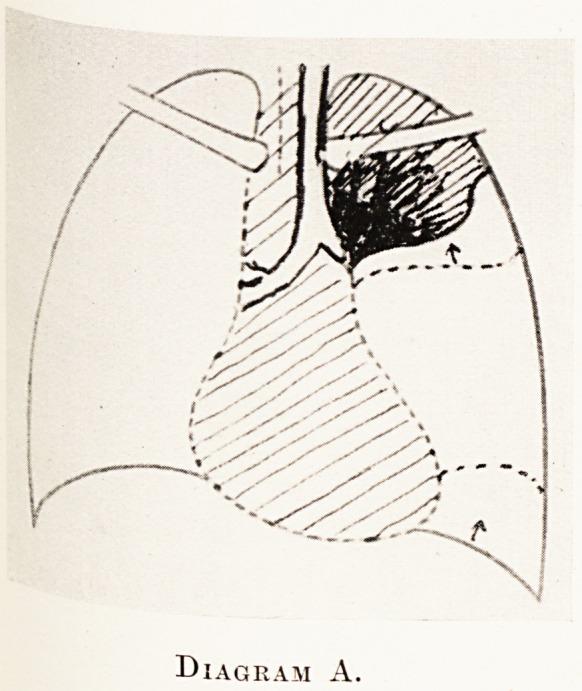


**Diagram B. f2:**
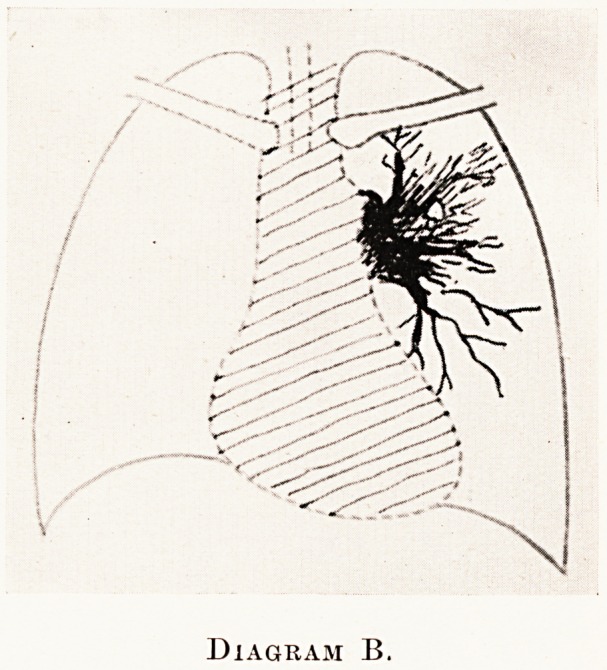


**Diagram C. f3:**
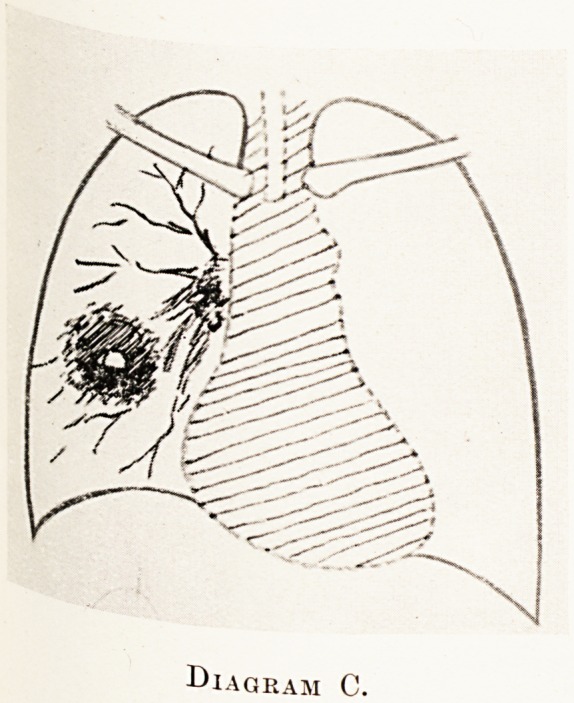


**Diagram D. f4:**
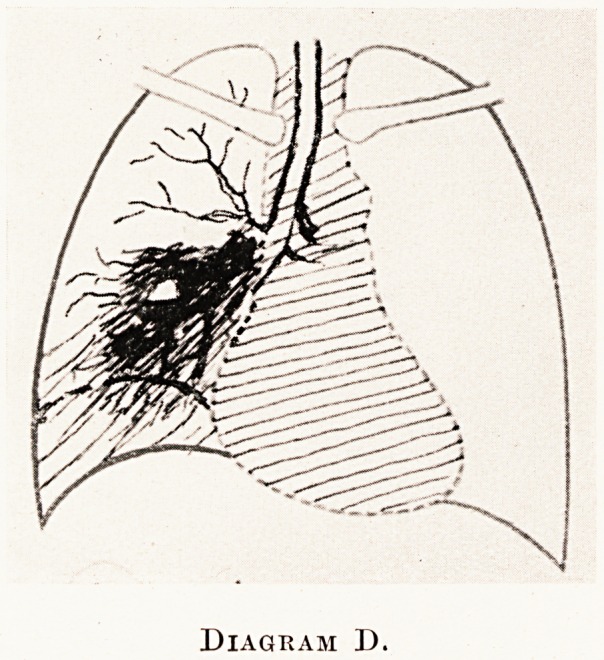


**Fig. 1. f5:**
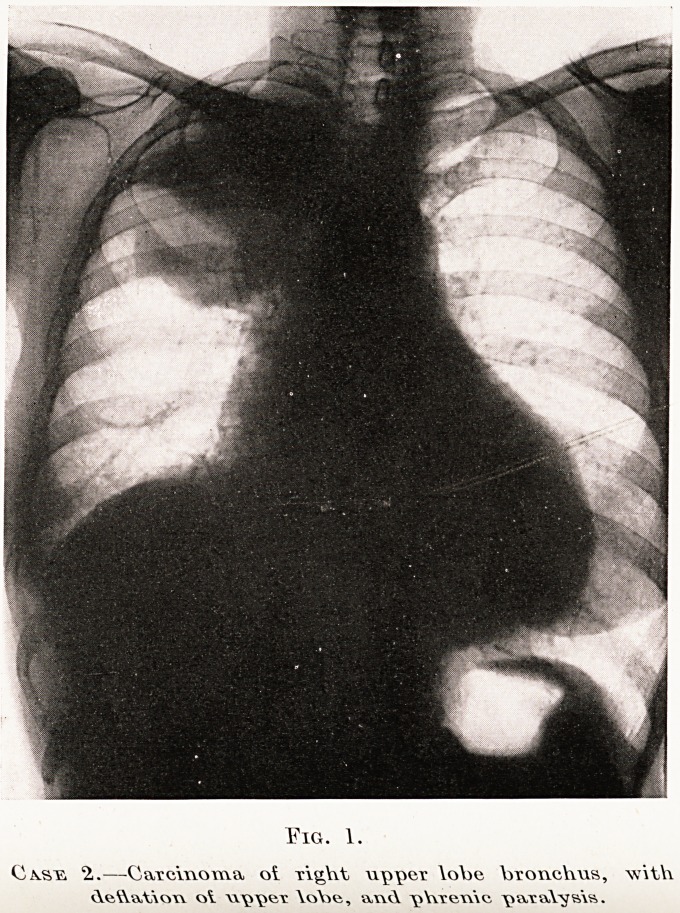


**Fig. 2. f6:**
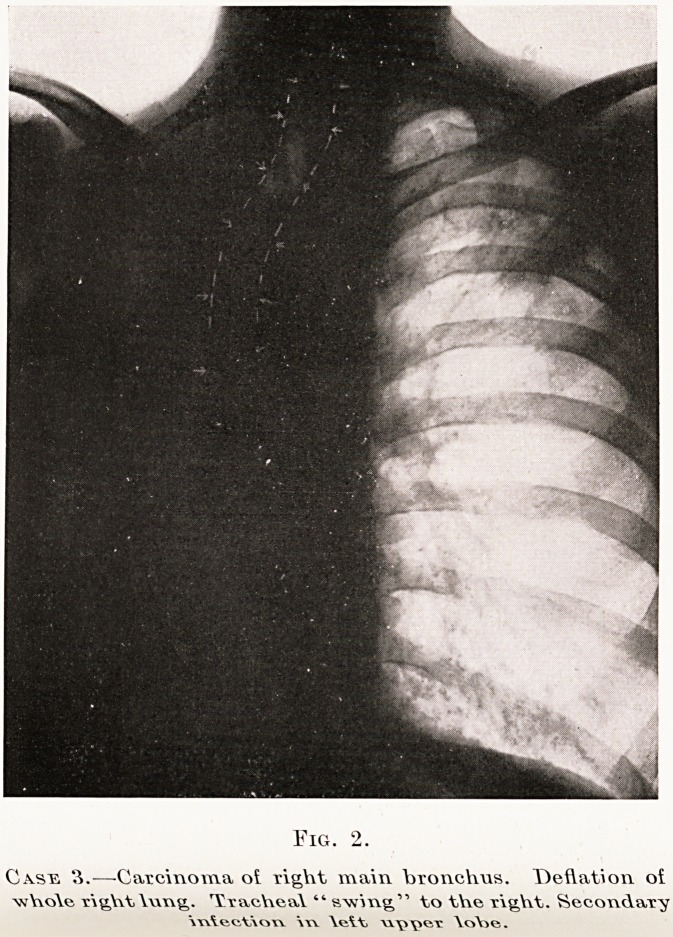


**Fig. 3. f7:**
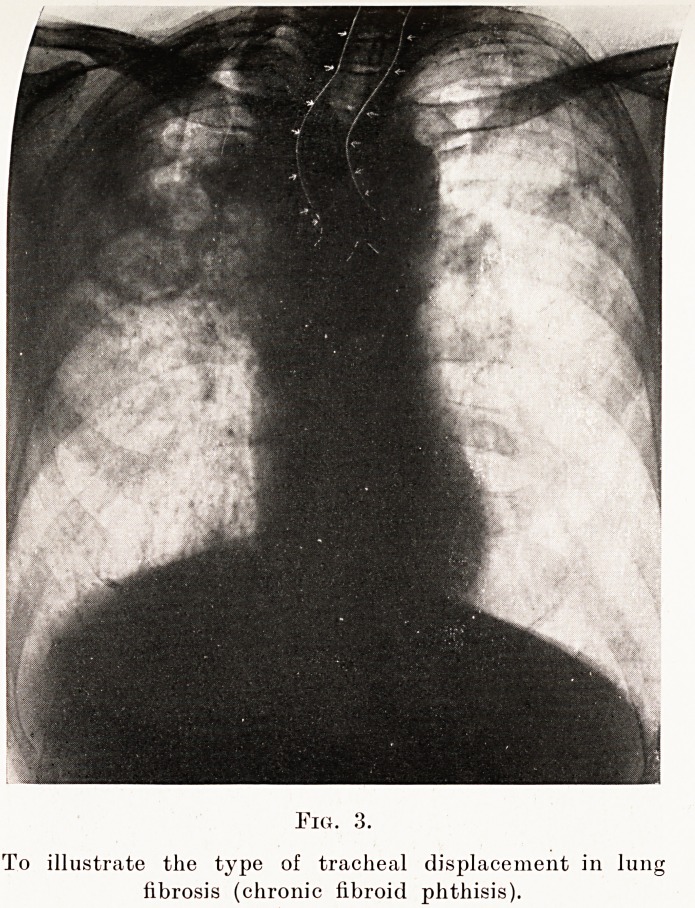


**Fig. 4. f8:**
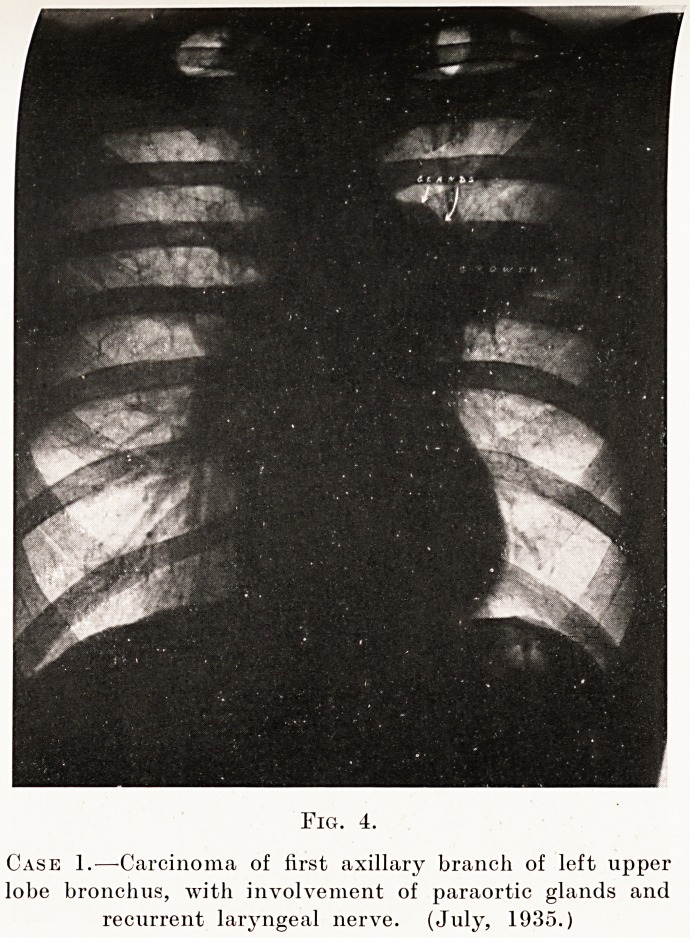


**Fig. 5. f9:**
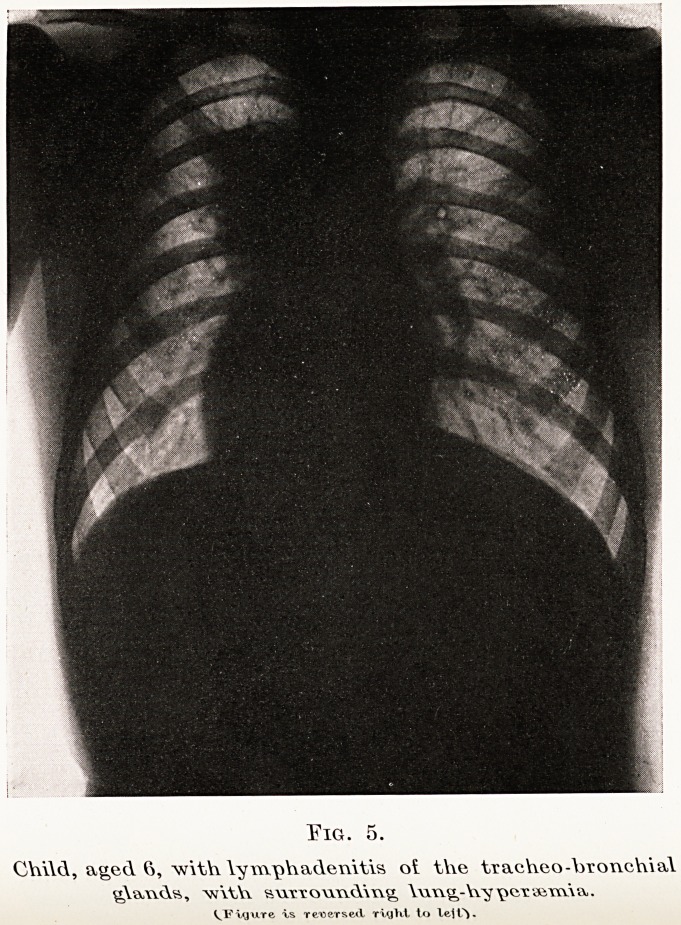


**Fig. 6. f10:**
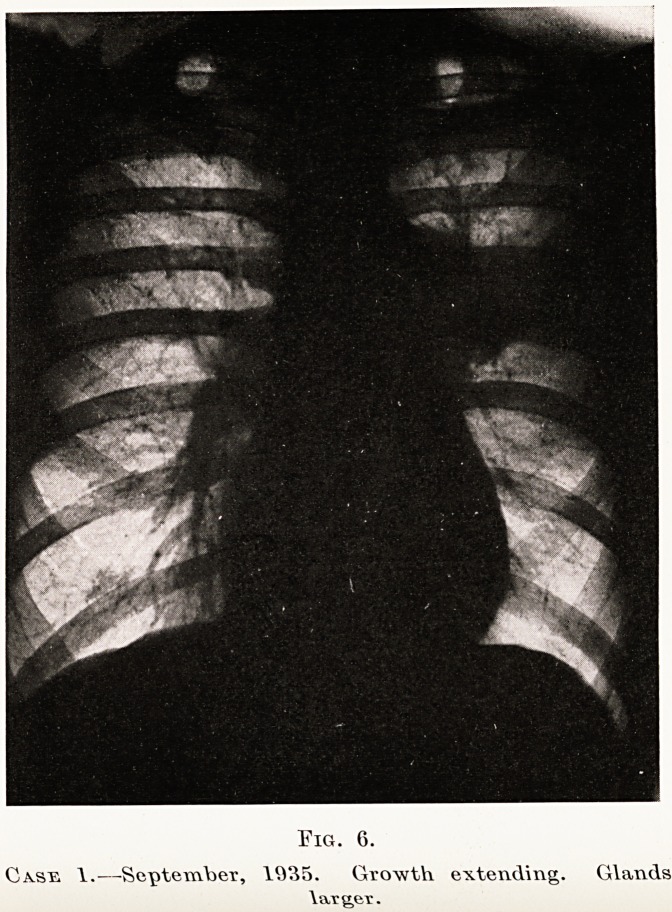


**Fig. 7. f11:**
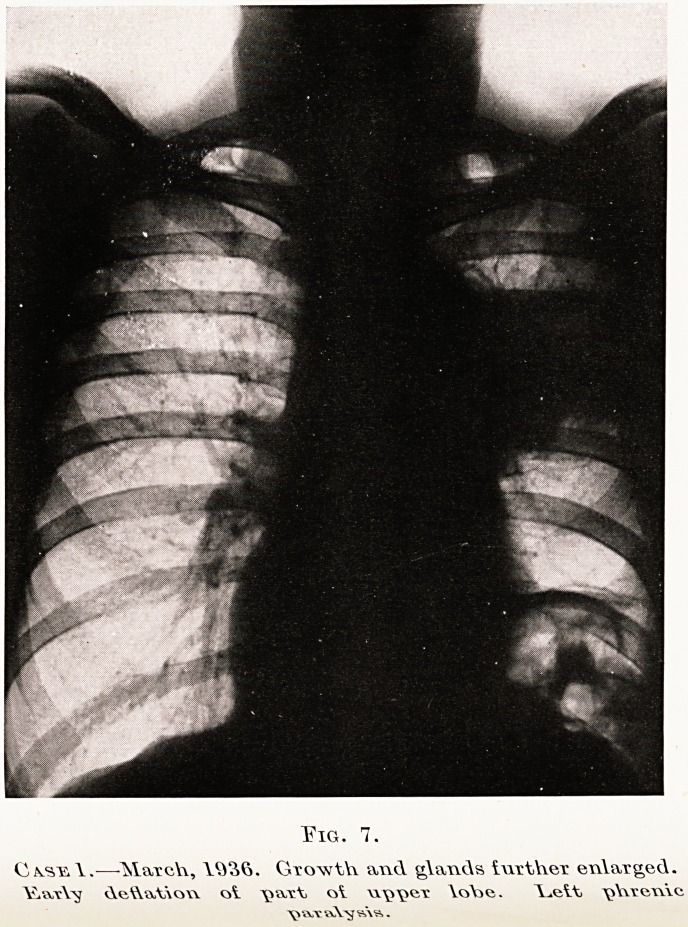


**Fig. 8. f12:**